# OIT3 serves as a novel biomarker of hepatocellular carcinoma by mediating ferroptosis *via* regulating the arachidonic acid metabolism

**DOI:** 10.3389/fonc.2022.977348

**Published:** 2022-09-05

**Authors:** Jie Wen, Abudureyimujiang Aili, Yao Xue Yan, YuLin Lai, Shaoqing Niu, Shasha He, Xiaokai Zhang, Guixiong Zhang, Jiaping Li

**Affiliations:** ^1^ Department of Interventional Oncology, The First Affiliated Hospital of Sun Yat-sen University, Guangzhou, China and Department of Medical Oncology and Radiation Sickness, Peking University Third Hospital, Beijing, China; ^2^ Department of Medical Oncology and Radiation Sickness, Peking University Third Hospital, Beijing, China; ^3^ Department of Dermatology, Peking University People’s Hospital, Beijing, China; ^4^ Deparment of Radiotherapy, The First Affiliated Hospital of Sun Yat-sen University, Guangzhou, China; ^5^ Department of Interventional Oncology, The First Affiliated Hospital of Sun Yat-sen University, Guangzhou, China

**Keywords:** HCC, OIT3, ferroptosis, arachidonic acid, biomarker

## Abstract

**Background:**

Oncoprotein-Induced Transcript 3 Protein (OIT3) was identified as a liver-specific gene with abnormal expression in hepatocellular carcinoma (HCC). Herein, we aimed to examine the function and specific mechanism of OIT3 in HCC.

**Methods:**

Bioinformatic analyses and tissue microarray *via* immunohistochemistry were used to validate the expression of OIT3 in HCC. The biofunctions of OIT3 in HCC were determined *in vitro* and *in vivo*. The mechanism was confirmed by RNA-Sequence and Western blotting. The uni- and multivariate analyses were used to identify the independent predictors for HCC.

**Results:**

Low expression of OIT3 was observed in HCC and predicted a poor clinical outcome. Ectopic expression of OIT3 could inhibit the proliferation, migration, and invasion abilities of HCC cells. Mechanistically, OIT3 upregulated the expression of ALOX15 and CYP4F3, thus inducing arachidonic acid increase, ROS accumulation, and lipid peroxidation, and eventually causing ferroptosis. OIT3 was validated as a prognostic predictor for HCC patients.

**Conclusions:**

Our findings revealed a novel role of OIT3 in the process of tumorigenesis of HCC. OIT3 inhibited reproliferation, migration, and invasion of HCC cells by triggering ferroptosis, which indicates that OIT3 could serve as a potential biomarker in HCC.

## Introduction

HCC is the sixth most common malignant tumor and the third cause of cancer-related death worldwide (1). A variety of factors can induce HCC; however, most HCCs are associated with hepatitis B virus infection, especially in China (2). Surgery is the first-line treatment for HCC (3). Yet, due to the lack of early specific symptoms, most patients are diagnosed at the middle or advanced stage, thus missing the opportunity for surgical treatment (4). As a general understanding of HCC gradually improved and technology evolved, it was found that targeted therapy, chemotherapy, interventional therapy, radiotherapy, and immunotherapy could provide some clinical benefits; nonetheless, the overall survival still remains unsatisfactory. In order to further improve the efficacy and diagnostic accuracy of HCC, it is important to thoroughly investigate the mechanisms involved in HCC and discover new biomarkers.

Ferroptosis is an iron-dependent form of cell death that catalyzes the liposome peroxidation of unsaturated fatty acids of the cell membrane, eventually leading to cell death (5). It has been associated with a variety of physiological and pathological processes. Previous studies have found that ferroptosis is important in treating cancer, including HCC (6). Sorafenib treatment in hepatocellular carcinoma causes ferroptosis in tumor cells by accumulating mitochondrial ROS and lipid peroxidation (7, 8). In addition, ferroptosis was found to be correlated with chemotherapy resistance and radiotherapy response in lung cancer (9). In ovarian cancer, ferroptosis was found to be related to aberrant ROS production and mitochondrial alterations (10).

Ferroptosis is a programmed cell death that is strictly regulated by various factors, such as the expression of GPX4 (11, 12), the import of iron (13), and the system of xCT (14). Since lipid peroxidation accumulation ultimately determines ferroptosis, lipid metabolism has an essential role in ferroptosis. Polyunsaturated fatty acids (PUFAs) are straight-chain fatty acids possessing 18 ~ 22 carbon atoms and two or more double bonds, rendering them more vulnerable to the attack by reactive oxygen species (ROS) for the production of lipid ROS (15). PUFAs usually include two series of fatty acids: omega-6 and omega-3 series. Arachidonic acid (AA) (C20:4) is a typical kind of omega-6 fatty acid (16). The lipid peroxidation can be triggered by ROS and the lipoxygenases, such as Arachidonate-15-Lipoxygenase (ALOX15) (17). ALOX15 is considered to be related to lipid-ROS production and to act as the mediator of ferroptosis in gastric cancer (18). It has been reported that CYP4F3, which is the main catalyst in the oxidation of fatty acid epoxides, is involved in the oxidation of important cellular mediators, including leukotriene B4 (LTB4) (19). The formation of leukotriene is detrimental to the cell proliferation of rat basophilic leukemia cells *via* ferroptosis-induced effects (20). Previous studies have also revealed that genes such as NRF2, P53, and Rb1 can regulate the process of ferroptosis in HCC (21–23). However, other factors may be involved in regulating ferroptosis in HCC.

Oncoprotein-Induced Transcript 3 Protein (OIT3) has been found to be downregulated in HCC patients and HCC cell lines. It was also identified as a liver-specific gene encoding the liver-specific Zona pellucida domain-containing protein (LZP) being mainly located on the nuclear membrane (24). The ZP domain has been recognized in many receptor-like eukaryotic glycoproteins with the regulation function of some important biological processes, including differentiation and signal transduction (25). OIT3 was found to be correlated with calcium ion binding to maintain the homeostasis of cellular Ca^2+^ and to be regulated by several transcription factors, including STAT, SOX9, and NF-Ka. OIT3 was reported to have copy number losses and low expression in colorectal cancer with uncertain biological function (26). OIT3 deficiency can promote the degradation of apoB and the accumulation of triacylglycerol in the liver, thus suggesting that OIT3 has an important role in lipid metabolism regulation (27).

Although OIT3 expression is decreased in HCC, its role in HCC and the corresponding molecular mechanisms have not been well investigated. In the present study, OIT3 significantly inhibited the growth of tumor cells *in vitro* and *in vivo*. We investigated the mechanism and found that OIT3 enhanced the ferroptosis by upregulating ALOX15 and CYP4F3 to mediate arachidonic acid increase, ROS accumulation, and lipid peroxidation. Our results revealed that OIT3 could act as a new regulator of ferroptosis and a potential therapeutic target in HCC.

## Methods

### Bioinformatics analysis

To determine the genes with obvious expression differences between HCC tissues and adjacent normal tissues, we downloaded the various datasets with the gene expression patterns, including GSE121248, GSE45050, GSE33006, GSE45267-young patients, and GSE45267-old patients. The genes with |logFC| > 1 and p-value < 0.05 were considered as the significantly differentially expressed genes (DEGs). The TOP 20 DEGs in each GSE dataset were selected as candidates according to the |logFC| values, after which the overlapping genes were further confirmed *via* Venn analysis. The OIT3 expression level in HCC tissues and normal tissues was verified through several online databases, including the GEPIA database (28) (http://gepia.cancer-pku.cn/index.html), GEO database (29) (https://www.ncbi.nlm.nih.gov/gds), and Ualcan database (30)(http://ualcan.path.uab.edu/). The associations between OIT3 expression and prognosis of HCC patients were validated using the following cancer databases: Kaplan-Meier plotter database (31) (http://kmplot.com/analysis/), Linkedomics database (32) (http://www.linkedomics.org/login.php) and HCCDB database (33) (http://lifeome.net/database/hccdb/home.html). The more detailed information is shown in **Table S2** in the additional file.

### Immunohistochemistry

Tumor tissue samples were removed from the nude mice and fixed with 4% paraformaldehyde for 24 hours, after which they were dehydrated and embedded to make 5μm thick sections. The sections were further deparaffinized, hydrated, and subjected to antigen retrieval in the sodium citrate buffer (10 mmol/L, pH 6.0) at 95°C for 15 minutes. Next, the sections were incubated with H_2_O_2_ solution (5%) at room temperature for 10 minutes, washed with phosphate buffer saline three times, and incubated with the 10% goat serum at room temperature for 15-20 minutes to block the unspecific binding sites. Subsequently, sections were incubated with primary antibodies, including OIT3, GPX4, Ki-67, ALOX15, and CYP4F3 at 4°C overnight and then analyzed by the PV-9000 2-step plus Poly-HRP anti-Mouse/Rabbit IgG Detection system (Zhong Shan Jin Qiao, China), after which the sections were developed with DAB solution and counter-stained with hematoxylin. The images were captured with Kfbio Slide Viewer (KONFOONG BIOTECH INTERNATIONAL CO., LTD). The detailed information on antibodies is listed in **Table S1** in the additional file.

The quantitation of histological scores was performed by three independent researchers who were blind to the detailed information of the studies. Scores representing the proportion of positive staining cells were divided into five grades as follows: 0 points (<10%); 1 point (11%-25%); 2 points (26%-50%); 3 points (51%-75%) and 4 points (>75%). The intensity of staining was scored as: 0 points (no staining); 1 point (weak staining); 2 points (moderate staining); and 3 points (strong staining). The total score was calculated as the result of the staining intensity score × positive cells score, including 0, 1, 2, 3, 4, 6, 8, 9, and 12 points.

### Tissue microarray

A tissue microarray containing 89 pairs of HCC tissues and the corresponding adjacent normal tissues collected by Outdo Biotech Co, Ltd (Shanghai, China) was used to validate the OIT3 expression levels in human HCC samples. Also, the detailed clinical characteristics of the 89 patients are listed in **Table S3**. Next, immunohistochemistry (IHC) was used to detect the expression of OIT3, after which the microarray slide was scanned with the PANNORAMIC slice scanner (3DHISTECH, PANNORAMIC DESK/MIDI/250/1000) to obtain the images for further analysis of the average optical density (AOD) *via* Indica labs system (U.S.A, Halo v3.0.311.314). According to the results shown in **Table S4**, the AOD of the HCC tissues ranged from 0.066586 to 0.103224; however, the AOD of adjacent normal tissues ranged from 0.08 to 0.18. The expression of OIT3 in HCC was divided into low expression (AOD ≤ 0.077457) and high expression (AOD>0.077457) groups.

### Cell culture

The human HCC cell lines HepG2 and Huh7 were kindly provided by professor Audureyimujiang Aili. Cells were cultured in Dulbecco Modified Eagle Medium (DMEM, Corning) supplemented with 10% fetal bovine serum (FBS, Excell Bio, FSP500) and 1% antibiotic-antimycotic at 37°C in a humidified atmosphere of 5% CO_2_.

### Extraction of total RNA and RT-qPCR assay

The TRIzol reagent (Invitrogen, Cat. No.10296-010) was applied to get total RNA from HCC cells with the guidance of the product manual. In brief, the total RNA was obtained, and the FastKing gDNA Dispelling RT SuperMix (TIANGEN, China) was used to synthesize the cDNA for mRNA. The Talent qPCR PreMix (SYBR Green)(TIANGEN, China) was used to detect the mRNA expression of OIT3, ALOX15, and CYP4F3; GAPDH was used as an internal control. The mRNA cDNA synthesis program was as follows: 42 ˚C for 15 min followed by 95 ˚C for 3 min. The mRNA detection program was as follows: 95 ˚C (15 min) for one cycle, followed by 40 cycles (10 s at 95°C, 32 s at 66°C), 10 s at 95 ˚C. The 2 ^−ΔΔCT^ mean was carried out to calculate the value of mRNA expression level. The qRT-PCR analysis was performed with QuantStudio 5. The detailed information on the primers is listed below.

OIT3 forward primer (5’ to 3’): GCGCCATTGAAGTGAGTGTC
OIT3 reverse primer (5’ to 3’): CAGGTTGGGCACGTATCCTT
ALOX15 forward primer (5’ to 3’): GTGGAAAACAGTGTGGCCAT
ALOX15 reverse primer (5’ to 3’): AGTAAGGTCCCAGGTGATGC
CYP4F3 forward primer (5’ to 3’): CAACCCCCGAAACGGAATTG
CYP4F3 reverse primer (5’ to 3’): TTCCCCGAGCTGTGAATCAG
GAPDH forward primer (5’ to 3’): GGAGCGAGATCCCTCCAAAAT
GAPDH reverse primer (5’ to 3’): GGCTGTTGTCATACTTCTCATGG

### Western blotting

The RIPA lysis buffer was used to extract the total protein of HCC cells. BCA assay was applied to detect the concentration of the protein of samples. Next, 30μg protein was loaded into the sodium dodecyl sulfate-polyacrylamide gel (SDS-PAGE) for electrophoretic separation, after which the protein in the gel was transferred to the polyvinylidene fluoride membrane (PVDF, Millipore 0.22um #IPVH 000 10), and the electric transferring time was dependent on the weight of the target proteins. Then, the PVDF membrane was blocked with the 3% blocking buffer and rocked gently at room temperature for 2 hours. The blocking buffer was then poured off, and the membrane was briefly rinsed with TBS buffer three times, 5 minutes each time. Then, samples were incubated with the first antibodies, including OIT3 (abbexa company, Catalogue No: abx103361, application concentration:1:1000), GPX4 (Cell Signaling Technology company, Catalogue No: #52455, application concentration:1:1000), ALOX15 (Bioss company, Catalogue No: bs-34007R, application concentration:1:1000), and CYP4F3 (Bioss company, Catalogue No: bs-14160R, application concentration:1:500) at 4°C overnight. The membrane was then gently washed with TBS buffer three times and incubated with second antibodies, including Anti-rabbit/mouse IgG, and HRP-linked antibody (1:2000, Beyotime Company) at room temperature for 2 hours. Finally, the membranes were exposed to get the blot images with the 1X SignalFire™ ECL Reagent (Millipore Company). The detailed information on antibodies is listed in **Table S1**.

### Establishment of the OIT3 stable overexpression HCC cell line

The OIT3-overexpressed lentivirus vector (OIT3-OE) and the corresponding control group (OIT3-OE ctrl) vector were purchased from Beijing Syngentech Co., LTD. The lentivirus vectors were transfected into HepG2 and Huh7 cells following an appropriate ratio of HCC cell number to lentivirus number described in the manufacturer’s instruction. After transfection for 48 h, the cells were placed in a medium containing puromycin of 3μg/ml for 72 hours. The cells that survived had stable overexpression of OIT3, after which the overexpression efficiency was validated by Western blotting. The gain-of-function analyses were performed in the HepG2 and Huh7 cells with OIT3 overexpression of the second to tenth passages.

### CCK8 assay

The transfected HCC cells were seeded in the 96-well plates with a density of 4000 cells/well, after which the CCK8 kit (Dojindo Laboratories, Japan) was applied to detect the cell proliferation ability. Briefly, 100μL DMEM containing 10μL CCK8 reagent was added into each well at 0 h, 24 h, 48 h, and 72 h after transfection. The optical density (OD) at 450 nm was tested with a multilabel plate reader (Thermo Fisher Scientific) after 2 hours of incubation.

### Colony formation assay

The transfected HCC cells were seeded in the 6-well plates with a density of 150 cells/well. Next, the cells were cultured for 14 days to form colonies. The methanol was used to fix the colonies at room temperature for 20 minutes, after which the methylene blue was used to stain the colonies. The colonies containing more than 50 cells were counted under a microscope.

### Migration and invasion assay

The transwell chamber inserts (8 mm, Corning) were used to measure the migration and invasion ability of HCC cells. For detection of invasion ability, matrigel (200 mL) was coated onto the upper compartment at 37°C for 4 hours. For migration ability detection, 5000 cells were resuspended in 200μL DMEM containing 1% FBS and seeded into the upper chamber. As for the invasion assay, 15000 cells were seeded into the upper chamber, and 1 mL DMEM containing 10% FBS was placed in the bottom chamber. After 24 hours, the non-migrated or invaded cells in the top chambers were removed, and the migrated or invaded cells at the lower chambers were fixed with 4% paraformaldehyde at room temperature for 20 minutes, after which they were stained with methylene blue at room temperature for 30 min. Next, the images of the cells were photographed using a microscope (Nikon, Japan, L300N/300ND).

### Wound healing assay

A wound healing assay was used to study the regulation of OIT3 expression on cell migration ability. The HepG2 and Huh7 cells with or without OIT3 overexpression were seeded in the 6-well plates, and the linear scratch wounds were made with a 200μl pipette tip when the confluence of cells≥90%. Each well was then gently rinsed with PBS to clear those floating cells, after which DMEM containing 3% fetal bovine serum and 1% antibiotic-antimycotic were applied to culture these cells for 48 hours. The scratch regions were photographed and measured at 0 and 48 hours. The ratio of wound closure (%) = the migrated area/the baseline area x 100%.

### ROS measurement

A ROS detection kit (Beyotime Biotechnology) was applied to measure the level of ROS in HCC cells. DCFH-DA was diluted with serum-free medium to 1:1000 so that the final concentration was 10μmol/L. Next, the medium was removed, and an appropriate volume of diluted DCFH-DA was added into the cell culture medium. The added volume was sufficient to cover the cells. Then, the cell was incubated at 37°C for 20 minutes and washed three times with a serum-free cell culture medium to fully remove the non-stained cells. Finally, a fluorescence microscope was used to observe and capture the image with the Ex/Em=488/525 nm.

### Lipid ROS measurement

A Lipid ROS kit (ABclonal, Catalog No.: RM02821) was used to detect the level of Lipid ROS in HCC cells. Briefly, 1mg of this product was dissolved in 198.2 μL DMSO to make the storage solution (10 mm). The storage was further diluted to the concentration of 10 μM and was then added to the cell culture medium for 1 hour of incubation. Next, the cells were washed twice with PBS to remove excess dye. They were harvested with trypsin and resuspended in a cell medium. The flow cytometry was used to detect the level of Lipid ROS. The mean of fluorescence signal ± SEM was used to quantify the level of the Lipid ROS in HCC.

### Malondialdehyde measurement

The levels of MDA in HCC cells were measured by an MDA detection kit (Solarbio life sciences company, BC0020). The HCC cells were harvested with trypsin into a centrifuge tube, after which 1 mL extract solution and the ultrasonic wave were used for crushing the cells at the following conditions: power 20%, ultrasonic 3s, interval 10s, repeated for 30 times. Next, the samples were centrifuged at 4°C, 8000g for 10 minutes, and the supernatant was collected. According to the product manual, 200μL mixed detection liquid and 100μL sample were added to the 96-well plates. The OD values of 450nm, 532nm, and 600nm were measured with a multilabel plate reader (Thermo Fisher Scientific). The blank group contained no samples in the wells. The calculation method was as follows:

ΔA450=A450 (sample)-A450 (blank)

ΔA532=A532 (sample)-A532 (blank)

ΔA600=A600 (sample)-A600 (blank)

MDA (nmol/mgprot) =5×(12.9×(ΔA532-ΔA600)-2.58×ΔA450)÷Spr (sample protein concentration, mg/mL).

### ELISA assay

The ELISA kit for arachidonic acid (AA, Catalog No.: RX100059H) was purchased from Ruixin Biotech company. The ELISA kits for LTB4 (Catalog No.: MM-1932H2), LTC4 (Catalog No.: MM-2107H2), LTD4 (Catalog No.: MM-0971H2), and LTE4 (Catalog No.: MM-0964H2) were bought from Meimian company. Following the manufacturer’s protocol, the procedure of the ELISA assay included standard sample dilution, sample addition, incubation, wash, enzyme addition, visualization, and detection.

### Mouse xenograft model

Balb/c male nude mice, 6-8 weeks old, weighing 20-25 g, were obtained from Vital River Laboratories, China. All the animals were housed in an environment with a temperature of 22 ± 1 °C, relative humidity of 50 ± 1%, and a light/dark cycle of 12/12 hr. All animal studies, including the mice euthanasia procedure were done in compliance with Peking University Third Medical School institutional animal care regulations and conducted according to the AAALAC and the IACUC guidelines (SA2022001). All animal experiments complied with the ARRIVE guidelines U.K. Animals (Scientific Procedures) Act, 1986 and associated guidelines and EU Directive 2010/63/EU for animal experiments.

For an exploration of the effect of OIT3 expression on the growth of HCC cells *in vivo*, the OIT3 overexpressed (OIT3-OE) and matching (OIT3-OE ctrl) HepG2 cells were subcutaneously injected. After 4 weeks, the mice were sacrificed, and the tumors were harvested. During this period, the size and weight of these tumors were recorded every four days. The cancer volume was calculated with the following formula: cancer volume (mm3) = (L × W^2^)/2, where L = long axis and W = short axis. The mice weight was measured during the experiment.and the mice weight change was calculated by the following formula: the mice weight change= the final weight-the original weight.

### RNA-sequence

Total RNA was harvested from the HepG2 cells in the OIT3-OE ctrl and OIT3-OE groups. The DEGseq V1.18.0 was used to analyze the DEGs between two groups. Genes with q ≤ 0.05 and |log2_ratio| ≥ 1 were considered as DEGs. Kyoto Encyclopedia of Genes and Genomes (KEGG) pathway analysis served to identify significantly enriched pathways with the help of the clusterProfiler (R package). The specific gene network of one pathway was generated based on the pathway topology analysis, and the study gene network was generated after mapping to the generated reference KEGG gene network. The Gene Ontology (GO) enrichment analysis, including Molecular Function (MF), Biological Process (BP), and Cellular Component (CC), was used for annotating genes and gene products by using GOseq (R package).

### Transmission electron microscopy

HepG2 and Huh7 cells of OIT3 overexpression and the corresponding control groups were harvested with trypsin, centrifuged at 250 x g for 10 min, and then fixed with 1% osmium tetroxide at 4˚C for 12 h. Subsequently, the cells were dehydrated with alcohol and acetone, embedded in epoxy resin, sectioned by an ultramicrotome, and stained with uranyl acetate and lead citrate. TEM images were captured with a transmission electron microscope (Japan, JEOL JEM-2100 Plus).

### Chelate iron detection

The FerroOrange kit (Dojindo, F347) was used for detecting intracellular Fe^2+^ levels in HCC cells. HepG2 and Huh7 cells of OIT3 overexpression and the corresponding control groups were seeded into the bottom of a black 96-well plate with a cell number of 10000 cells/well and incubated overnight. Next, the cells were washed with 100μL DMEM without FBS three times. 100μL FerroOrange Working Solution (1 μmol) was added to the wells and incubated for 30 min at 37°C, after which the fluorescence intensity of each sample was detected with a multifunctional enzyme labeling instrument.

### Statistical analysis

Data were generally shown as mean ± s.d. values. Statistical significance was determined using a two-tailed student’s t-test with GraphPad Prism 5. The 5 Prism 5aphPad Prism analyzed the associations between OIT3 expression and clinical characteristics. A p-value < 0.05 was considered statistically significant. The Kaplan-Meier method was carried out for survival analysis. The univariate and multivariable Cox proportional hazards regression were performed to identify the independent prognostic factors. The hazard ratio (HR) and 95% confidence interval (95% CI) were measured. HR > 1 indicated that gene expression was negatively associated with prognosis, while HR < 1 indicated a positive correlation.

## Results

### OIT3 is downregulated in HCC tumor tissues and positively associated with clinical outcomes

**Figure 1:**
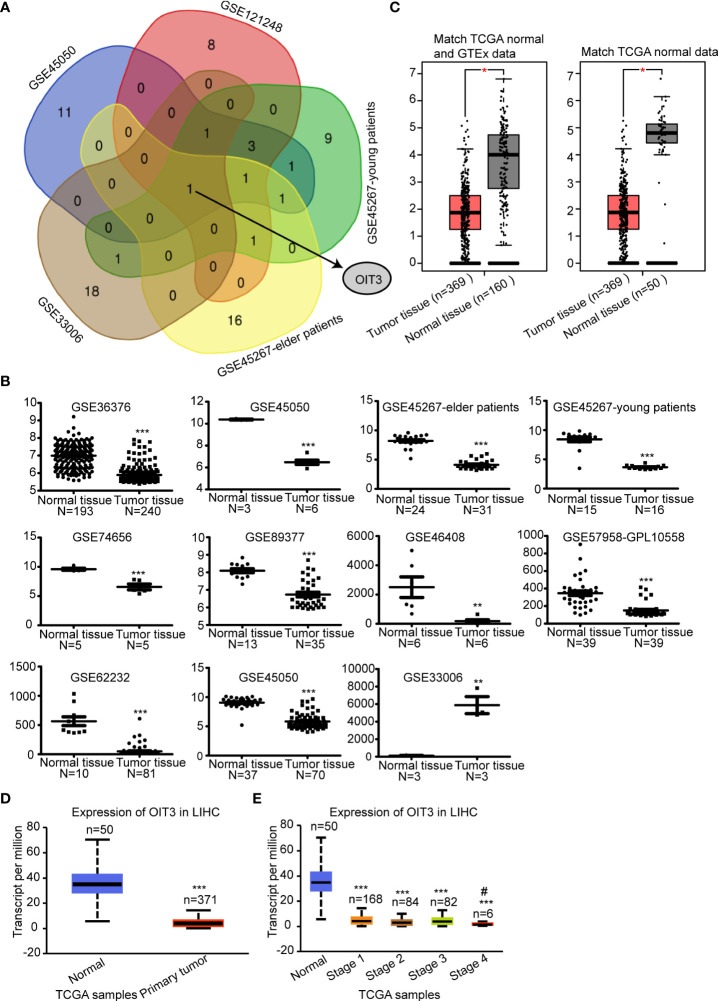
OIT3 was downregulated in HCC tissues. **(A)** OIT3 was a common differentially expressed mRNA among datasets, including GSE45050, GSE121248, GSE33006, GSE45267-elder patients, and GSE45267-young patients. **(B)** The expression levels of OIT3 in different GSE databases, including GSE36376, GSE45050, GSE45267-elder patients, GSE45267-young patients, GSE74656, GSE89377, GSE46408, GSE57958-GPL10558, GSE62232, GSE45050 and GSE33006. (**P < 0.01, ***P < 0.001). **(C)** OIT3 expression level in tumor tissues and normal tissues of HCC patients in the GEPIA database. The gene expression profiles of OIT3 from all available samples classified as LIHC tumor tissues and normal tissues in TCGA and GTEx were chosen to perform differential analysis in GEPIA. (*P < 0.05). **(D)** OIT3 expression level in tumor tissues and normal tissues of LIHC patients in Ualcan database (***P < 0.001). **(E)** OIT3 expression in HCC patients at different stages of LIHC patients in Ualcan database (AJCC stage. stage 1, stage 2, stage 3 and stage 4 group compared with the normal group, ***P < 0.001. Stage 3 group compared with stage 4 group, *^#^P < 0.05*). LIHC, Liver hepatocellular carcinoma.

The gene expression characteristics of HCC tumor tissues and paracancerous tissues were analyzed. After analyzing the tumor and paracancerous tissue, 20 genes with the most significant differences were selected. Next, the overlapped mRNAs were screened, revealing OIT3 as the only potential target (**Figure 1A** and **Table S2**). Meanwhile, we checked the expression level of OIT3 in various GSE datasets of HCC (**Figure 1B**), finding that OIT3 had a generally lower expression level in HCC tissues than that in the normal tissues, except for GSE33006 (**Figure 1B**). We also verified the downregulated expression level of OIT3 in TCGA HCC samples *via* the GEPIA database (**Figure 1C**) and the UALCAN database (**Figure 1D**), finding that compared with early HCC, advanced tumors had lower expression of OIT3 (**Figure 1E**), indicating that OIT3 had a significant clinical relevance.

**Figure 2:**
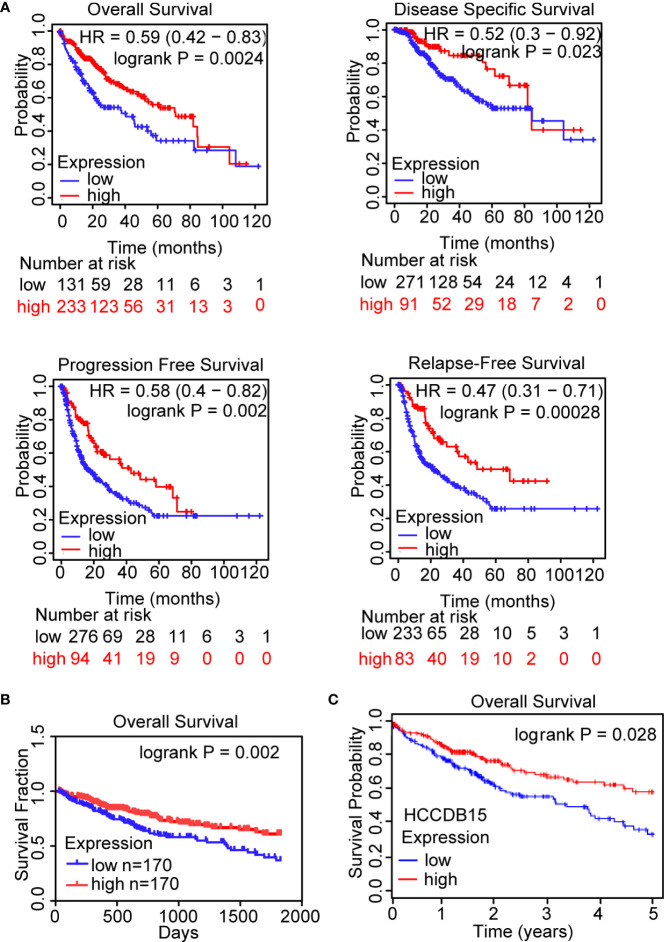
OIT3 expression was positively associated with the prognosis of HCC patients. **(A–C)** The clinical outcomes were markedly decreased in HCC patients with low OIT3 expression according to the Kaplan-Meier plotter database **(A)**, Linkedomics database **(B)**, and HCCDB database **(C)**. HR, hazard ratio.

To investigate whether OIT3 could act as a biomarker in the prognosis of HCC patients, Kaplan-Meier analysis was carried out in the Kaplan-Meier plotter database (**Figure 2A**), Linkdomeci database (**Figure 2B**), and HCCDB database (**Figure 2C**). Patients with higher OIT3 expression levels were found to have a better probability of OS (overall survival), DFS (disease-free survival), PFS (progression-free survival), and RFS (relapse-free survival) (**Figure 2A**). Besides, the data in the Linkdomeci database (**Figure 2B**) and HCCDB database (**Figure 2C**) showed the same trend.

Our findings indicated that OIT3 was downregulated in HCC patients, and its expression level was positively associated with clinical outcomes.

### OIT3 is an independent prognostic factor of HCC patients

**Figure 3:**
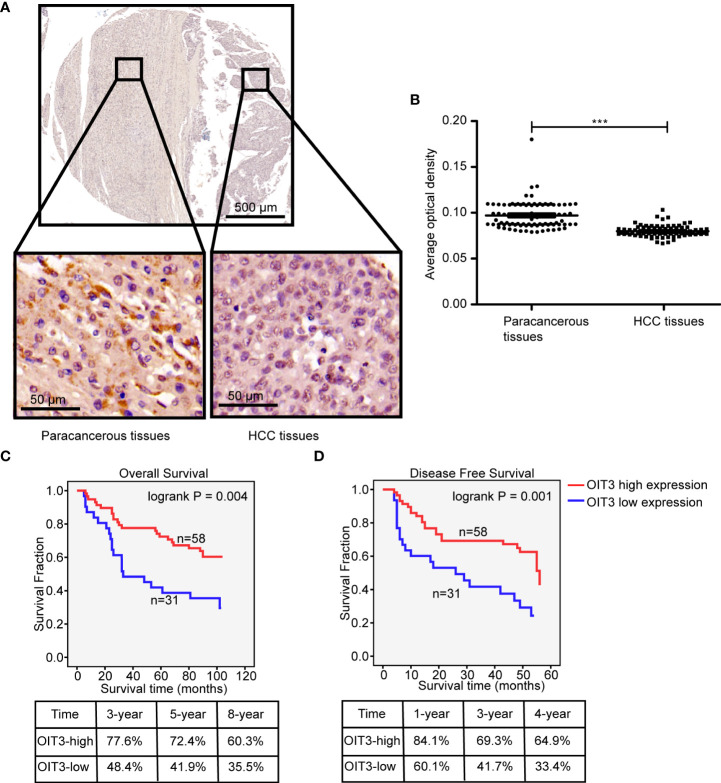
OIT3 was lowly expressed in HCC tissues and significantly correlated with clinical outcomes. **(A–B)** OIT3 was downregulated in HCC tissues, as validated using ISH with a tissue microarray. As shown, HCC tissues with richer cytoplasms and larger nuclei had weaker OIT3 staining than that in the paracancerous tissues **(A)**, accompanied by the statistical result **(B)** (n= 89, ***P < 0.001). **(C)** The clinical outcomes of HCC patients with the different expression levels of OIT3. The 3-, 5-, and 8-year overall survival rates of the OIT3 high group were 77.6%, 72.4%, and 60.3% vs. 48.4%, 41.9%, and 35.3% of the OIT3 low group, respectively. **(D)** The 1-, 3-, and 4-year disease-free survival rates of the OIT3 high group were 84.1%, 69.3%, and 64.9% vs. 60.1%, 41.7%, and 33.4% of the OIT3 low group, respectively.

In order to assess the clinical correlation of OIT3 expression in HCC, we measured the OIT3 protein expression level through IHC examination using a tissue microarray, which contained HCC tissues and the corresponding paracancer tissues of 89 patients. As shown in **Figures 3A, B**, IHC analysis validated the downregulated OIT3 expression in HCC tissues, revealing the lower proportion score and staining intensity score of positive cells (**Figures 3A, B**). In addition, HCC patients with higher OIT3 expression levels had better overall survival rates (OS, 8-year survival rates, OIT3-high group: 60.3% vs. OIT3-low group: 35.5%) and disease-free survival rates (DFS, 4-year survival rates, OIT3-high group: 64.9% vs. OIT3-low group: 33.4%) (**Figures 3C, D**). **Table S3** contains the clinical characteristics of the 89 HCC patients, including sex, diagnosis age, T stage, OIT3 expression status, and so on. As shown in **Table S5**, T stage, AJCC stage, and recurrence status were significantly correlated with OIT3 expression status. Those HCC patients at the T1 stage (P=0.021), AJCC I stage (P=0.042), and with free recurrence (P=0.018) had higher OIT3 levels.

**Figure 4:**
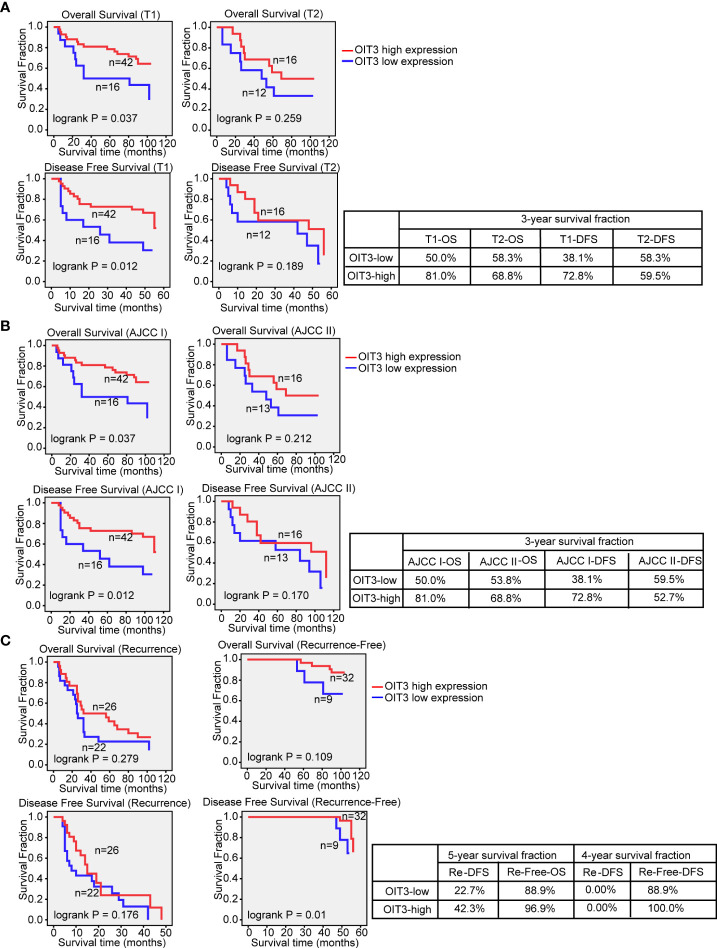
OIT3 was positively associated with HCC outcome at T1 and AJCC I stage. **(A)** The effect of OIT3 expression on the clinical outcomes of HCC patients at T1 and T2 stage. As shown, HCC patients at the T1 stage with a higher level of OIT3 had a better prognosis (3-year OS rates: OIT3 high group vs. OIT3 low group, 81.0% vs. 50.0%; 3-year DFS rates: OIT3 high group vs. OIT3 low group, 72.8% vs. 38.1%). **(B)** The effects of OIT3 expression on the clinical outcomes of HCC patients at AJCC I and AJCC II stage. As shown, HCC patients at AJCC I stage with a higher level of OIT3 had a better prognosis (3-year OS rates: OIT3 high group vs. OIT3 low group, 81.0% vs. 50.0%; 3-year DFS rates: OIT3 high group vs. OIT3 low group, 72.8% vs. 38.1%). **(C)** As shown, HCC patients with free recurrence and a higher level of OIT3 had a better prognosis (4-year DFS rates: OIT3 high group vs. OIT3 low group, 100.0% vs. 88.9%). OS, overall survival; DFS, disease-free survival; Re, recurrence.

Uni- and multivariate analyses were performed to further dissect the independent predictors for OS and DFS of the HCC. As shown in Table S6, T stage, AJCC stage, recurrence status, and OIT3 expression level were markedly correlated with OS and DFS. Moreover, tumor size had an obvious association with DFS. The HCC patients with higher OIT3 expression levels at the T1 stage and AJCC I stage had longer OS and DFS time. However, the OIT3 expression level only had a significant positive effect on the DFS time of recurrence-free patients (**Figures 4A–C**). In order to determine the independent prognostic factors, multivariate cox proportional analysis models of OS and DFS were carried out (**Table S7**), revealing the recurrence status as the independent predictor of OS and DFS. Compared to the patients without recurrence, those with recurrence had a higher risk of death (OS: HR=7.825, 95%CI=3.404-17.992; DFS: HR =166.575, 95%CI=19.834-1399.009). Also, compared to HCC patients with low OIT3 expression, those with high OIT3 levels had a better DFS (HR=0.493, 95%CI=0.257-0.946), suggesting that OIT3 expression level was independently associated with the DFS of HCC patients.

Taken together, we concluded that OIT3 was downregulated in HCC patients, and the higher OIT3 expression level predicted a better clinical outcome. Moreover, OIT3 was an independent prognostic factor for HCC patients.

### OIT3 inhibits the proliferation, clone formation, migration, and invasion abilities of HCC cells *in vitro*

**Figure 5:**
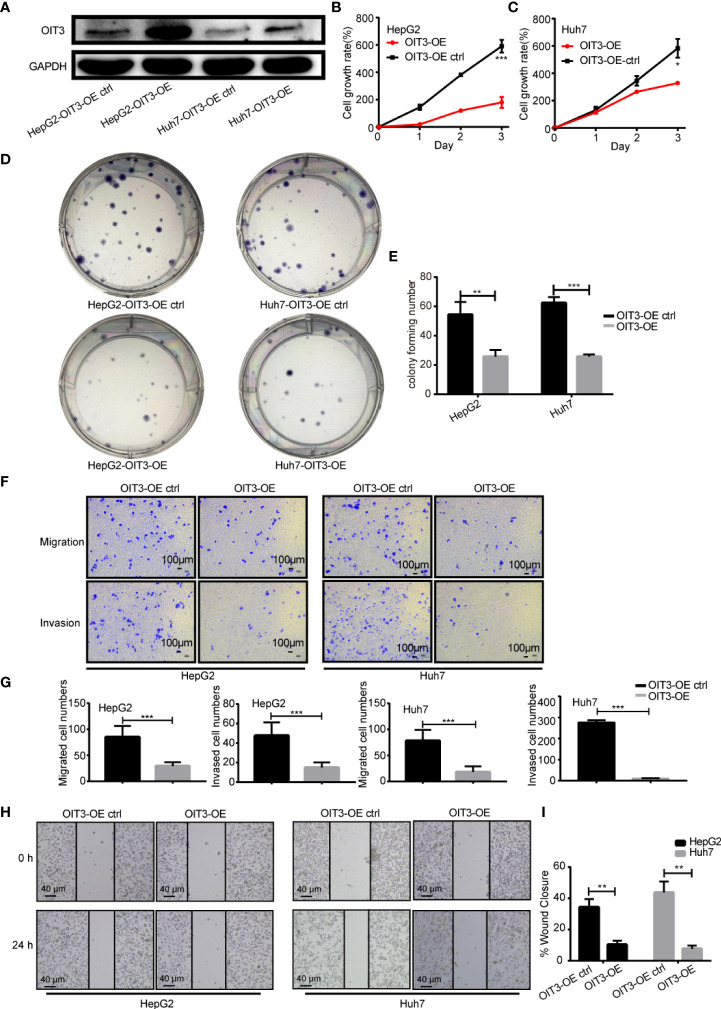
OIT3 suppressed the proliferation, clone formation, migration, and invasion abilities of HCC cells *in vitro*. **(A)** OIT3 protein level could be increased by OIT3-overexpressed lentivirus vector in HepG2 and Huh7 cells. **(B, C)** The overexpression of OIT3 could inhibit the proliferation abilities of HepG2 and Huh7 cells. (*P < 0.05, ***P < 0.001). **(D, E)** OIT3 upregulation could decrease the clone formation abilities of HepG2 and Huh7 cells. (*P < 0.05, **P < 0.01). **(F, G)** OIT3 upregulation could suppress the migration and invasion abilities of HepG2 and Huh7 cells. (***P < 0.001). **(H, I)** The effects of OIT3 expression on migration abilities of HepG2 and Huh7 cells were detected with a scratch wound-healing assay. OIT3’s overexpression showed inhibited wound closure levels of 10.3 ± 2.5% in HepG2 cells and 7.7 ± 2.1% in Huh7 cells compared with the control groups respectively (49.7 ± 4.5% in HepG2 cells and 22.7 ± 2.5% in Huh7 cells) for 48 h. Experiments were repeated three times, and the data are expressed as the mean ± SEM. OIT3-OE, OIT3-overexpressed lentivirus vector; OIT3-OE ctrl, OIT3-overexpressed lentivirus vector control.

Next, the biological functions of OIT3 in HCC cells were studied to further investigate the role of OIT3 in HCC. Since OIT3 was lowly expressed in tumor cells, we overexpressed OIT3 with a lentivirus vector in various HCC cell lines. As shown in **Figure 5A**, OIT3 was remarkably upregulated by the OIT3-overexpressed lentivirus vector (OIT3-OE) compared with the control group (OIT3-OE ctrl) in HepG2 and Huh7 cell lines. Moreover, the overexpression of OIT3 significantly decreased cell proliferation (**Figures 5B, C**), clone formation (**Figures 5D, E**), migration, and invasion abilities (**Figures 5F, G**) in HepG2 and Huh7 cell lines. The scratch wound-healing assay in **Figures 5H, I** showed that HepG2 and Huh7 cells with OIT3 overexpression migrated slower than the corresponding control group cells, thus implying that OIT3 acted as a tumor suppressor gene in HCC.

### OIT3 promotes the ferroptosis of HCC cells by increasing the expression of ALOX15 and CYP4F3 to mediated ROS, lipid-ROS accumulation, and arachidonic acid metabolism activation

**Figure 6:**
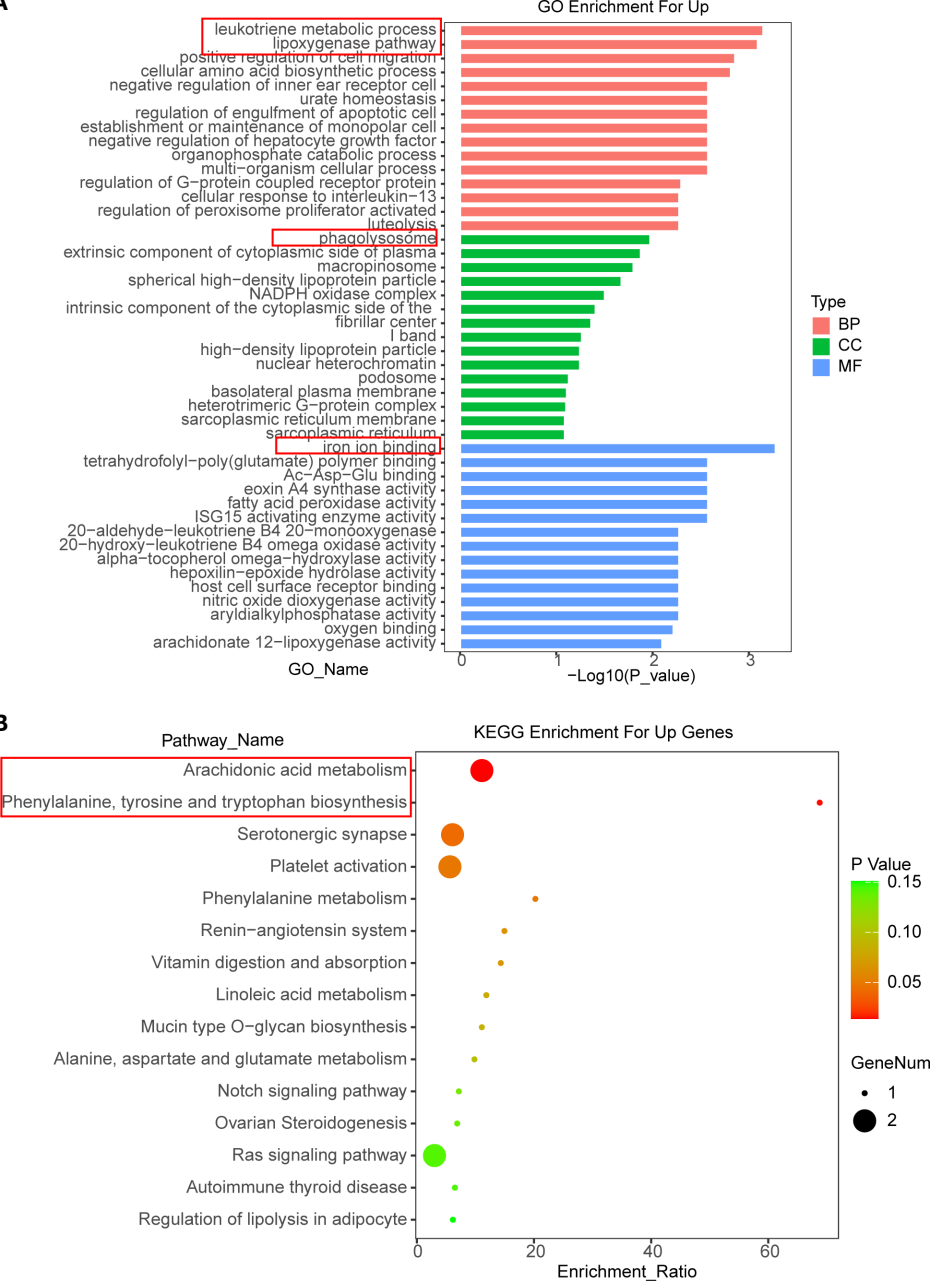
The functional annotation for the DEGs based on the DAVID. **(A, B)** The differently expressed genes of HepG2 cells related to biological process (BP), cellular component (CC), molecular function (MF), and KEGG enrichment analysis were displayed separately. BP, biological process; CC, cellular component; MF, molecular function.

In order to explore the regulatory mechanism of OIT3 in HCC, an RNA sequence was performed to discover the gene expression profile induced by OIT3. As shown in **Figures S1A, B**, the RNA sequencing data reported that 146 genes were overexpressed and 208 genes were downregulated in HepG2 cells after OIT3 upregulation, respectively. Thereafter, we conducted the Gene Ontology (GO) analysis, which revealed that the leukotriene metabolic process and the lipoxygenase pathway ranked in the top 2 in the biological process (BP) categories. Meanwhile, phagolysosome and iron ion binding ranked first in the cellular component (CC) and molecular function (MF) categories, respectively (**Figure 6A**). Consistent with GO analysis results, KEGG pathway analysis showed that these upregulated mRNAs were enriched in 15 signaling pathways, and the arachidonic acid metabolism and the phenylalanine, tyrosine, and tryptophan biosynthesis ranked as the top 2 pathways (**Figure 6B**).

**Figure 7:**
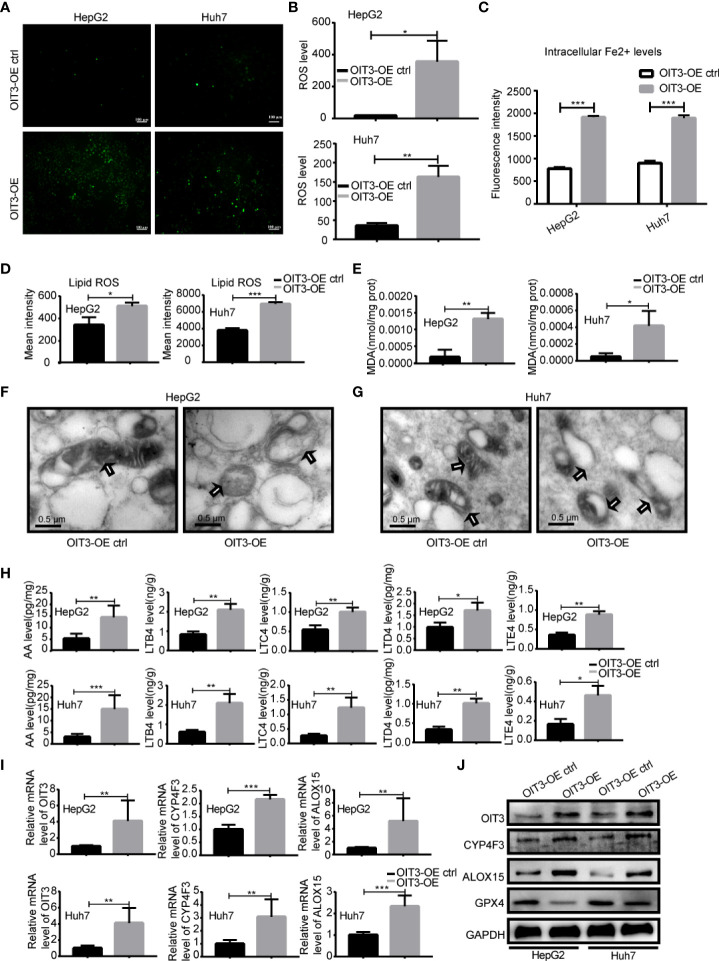
OIT3 promoted the ferroptosis of HCC cells *in vitro*. **(A, B)** The ROS level in HepG2 and Huh7 cells with different OIT3 expression. (*P < 0.05, **P < 0.01). **(C)** The intracellular Fe^2+^ level of HepG2 and Huh7 cells with different OIT3 expression. (***P < 0.001). **(D)** The lipid ROS level in HepG2 and Huh7 cells with different OIT3 expressions. (*P < 0.05, ***P < 0.001). **(E)** The effect of OIT3 expression on the production of MDA in HepG2 and Huh7 cells. (*P < 0.05, **P < 0.01). **(F, G)** Transmission electron microscopy revealed mitochondrial alterations in HepG2 and Huh7 cells with or without OIT3 overexpression (bar=0.5 μm). **(H)** OIT3 enhanced the level of AA, LTB4, LTC4, LTD4, and LTE4 in HepG2 and Huh7 cells. (*P < 0.05, **P < 0.01, ***P < 0.001). **(I)** OIT3 upregulated the expression of ALOX15 and CYP4F3 at mRNA level (**P < 0.01, ***P < 0.001). **(J)** The protein level of ALOX15, CYP4F3, and GPX4 in HepG2 and Huh7 cells with the different expression levels of OIT3. Experiments were repeated three times, and the data are expressed as the mean ± SEM. OIT3-OE, OIT3-overexpressed lentivirus vector; OIT3-OE ctrl, OIT3-overexpressed lentivirus vector control; AA, arachidonic acid; LTB4, leukotriene B4; LTC4, leukotriene C4; LTD4, leukotriene D4; LTE4, leukotriene E4.

According to various previous studies, leukotriene metabolism, lipoxygenase, phagolysosome, iron ion, arachidonic acid metabolism, and tryptophan could regulate ferroptosis (34–38). Therefore, we reasoned that OIT3 might be involved in the process of ferroptosis in HCC cell lines. As ferroptosis results from the catastrophic accumulation of ROS, we measured the potential changes of ROS in HCC cells after a change in OIT3 expression. As shown in **Figures 7A, B**, OIT3 upregulation significantly enhanced ROS production compared to the control group. Moreover, OIT3 overexpression caused a marked increase in intracellular Fe^2+^ levels, which was one of the key factors to induce ferroptosis (**Figure 7C**). We further verified that OIT3 overexpression mediated the sharp increase of lipid-ROS and MDA, which were the key factors and reactive products of ferroptosis, and indicated that OIT3 induced ferroptosis of HCC cells (**Figures 7D, E**). The effects of OIT3 on HepG2 and Huh7 cells were observed with electron microscopy. Moreover, our results indicated that in the OIT3 upregulation group, the small and atrophied mitochondria were seen, and the mitochondrial crista was reduced or even disappeared, producing a lot of empty bubbles. The mitochondrial rupture also happened, in line with the morphological characteristics of ferroptosis (**Figures 7F, G**). Considering that arachidonic acid (AA) is a 20-carbon unsaturated fatty acid, it can be metabolized in the body and converted into a series of active substances, including leukotrienes (LTs). Arachidonic acid and leukotrienes can be used as the reaction substrate of ferroptosis, and the level of AA, LTB4, LTC4, LTD4 as well as LTE4 could be markedly increased by OIT3 (**Figure 7H**). According to the RNA sequence results, the differentially expressed genes involved in the arachidonic acid metabolism pathway included ALOX15 and CYP4F3. Next, we confirmed that OIT3 indeed upregulated the expression of ALOX15 and CYP4F3 at both mRNA and protein levels (**Figures 7I, J**). Moreover, OIT3 overexpression led to the obvious reduction of the GPX4 protein level, suggesting that OIT3 could boost ferroptosis in HCC cells (**Figure 7J**).

**Figure 8:**
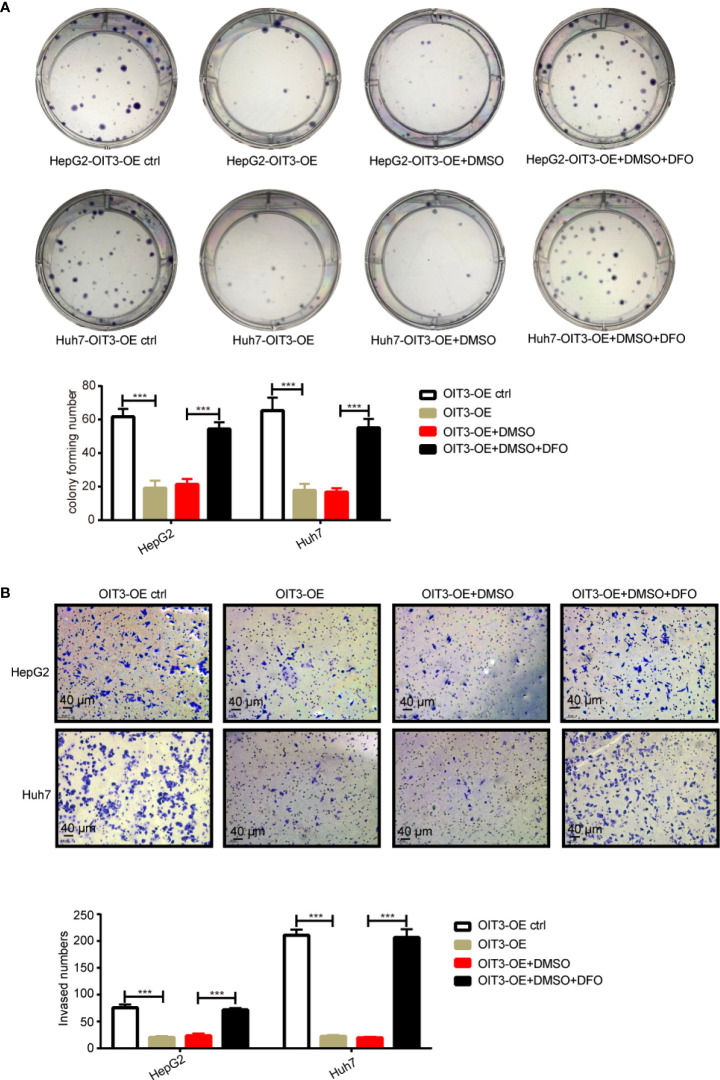
The OIT3 mediated-inhibition of clone formation and invasion abilities could be reversed by DFO. **(A)** The effect of DFO (100 μmol/L) on the clone formation ability of HepG2 and Huh7 cells. (***P < 0.001). **(B)** The effect of DFO (100 μmol/L) on the clone invasion ability of HepG2 and Huh7 cells. (***P < 0.001). Experiments were repeated three times, and the data are expressed as the mean ± SEM. OIT3-OE, OIT3-overexpressed lentivirus vector; OIT3-OE ctrl, OIT3-overexpressed lentivirus vector control; DFO, desferrioxamine; DMSO, dimethyl sulfoxide.

Furthermore, desferrioxamine (DFO), an iron chelator that inhibits the ferroptosis process, rescued OIT3 overexpression mediating the suppression of the phenotypic function of cells, including clone formation and invasion in HepG2 and Huh7 cell lines (**Figures 8A, B**). These results indicated that OIT3 upregulation restrains cell growth and motility abilities of HCC cells *via* ferroptosis.

### OIT3 suppresses the growth of HCC cells by inducing the ferroptosis process *in vivo*

**Figure 9:**
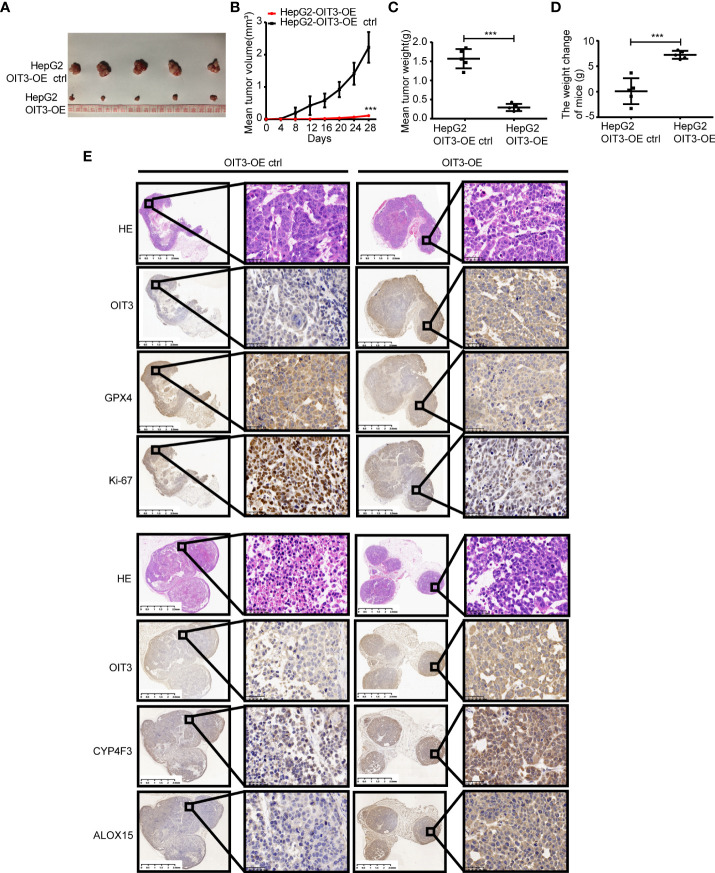
OIT3 promoted the ferroptosis of HCC cells *in vivo*. **(A–C)** The volume and weight of HCC tumors with different levels of OIT3 expression (n = 5, ***P < 0.001). **(D)** The weight change of mice in different groups.(n = 5, ***P < 0.001). **(E)** Compared to the control group, lower levels of GPX4 and Ki-67 with higher levels of ALOX15 and CYP4F3 could be observed in the mice from the OIT3 upregulating group. OIT3-OE, OIT3-overexpressed lentivirus vector; OIT3-OE ctrl, OIT3-overexpressed lentivirus vector control.

**Figure 10:**
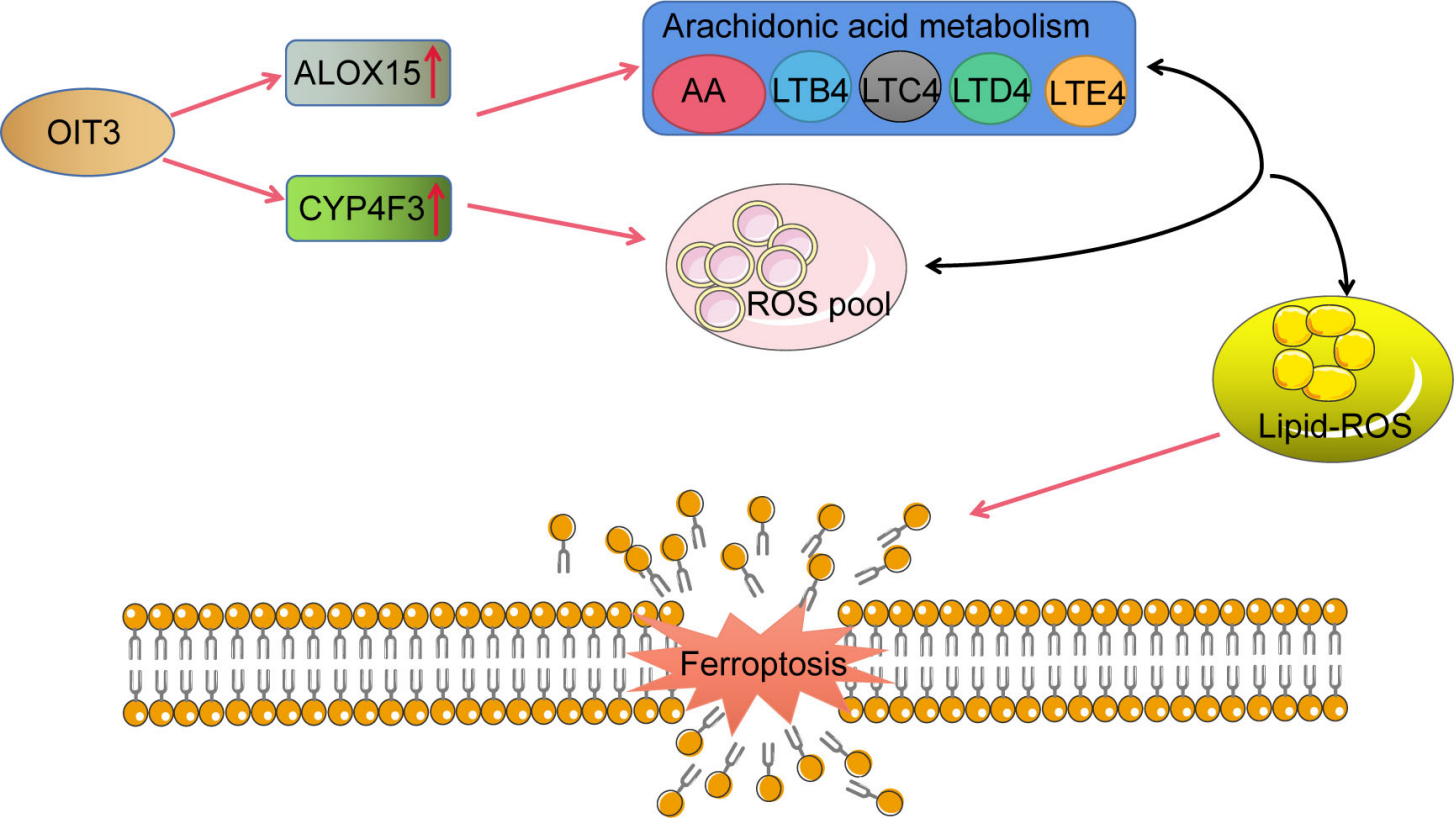
The schematic representation of OIT3 signaling in HCC. AA, arachidonic acid; LTB4, leukotriene B4; LTC4, leukotriene C4; LTD4, leukotriene D4; LTE4, leukotriene E4.

To investigate the effects of OIT3 on HCC growth *in vivo*, the HepG2 cells with the overexpression of OIT3 were implanted into the backpack of nude mice. Consistent with the results of *in vitro* experiments, OIT3 upregulation markedly inhibited the growth of HepG2 cells *in vivo* (**Figures 9A–C**). Compared to control mice, the body weight of mice was significantly improved by OIT3 upregulation (**Figure 9D**). Meanwhile, fewer Ki-67-positive cells were observed in tumor tissues from the OIT3 overexpression group compared to the control group (**Figure 9E**). Besides, OIT3 overexpression decreased the level of GPX4, indicating the activated ferroptosis process (**Figure 9E**). The expression levels of ALOX15 and CYP4F3 were also elevated by OIT3 (**Figure 9E**). In summary, as shown in the idiogram in **Figure 10**, OIT3 could upregulate the level of ALOX15 and CYP4F3, thus activating the arachidonic acid metabolism pathway to boost the production of arachidonic acid, leukotrienes (LTB4, LTC4, LTD4, and LTE4), and ROS. Arachidonic acid and leukotrienes are substances with high activity that can be used as reaction substrates for the ROS peroxidation process, eventually mediating the lipid ROS accumulation for ferroptosis.

## Discussion

Currently, there are several studies on the OIT3 gene in HCC. OIT3, also known as LZP, has been identified due to its downregulation in HCC tissues (39) and high expression in liver tissues (27). However, the physiological functions and molecular mechanisms of LZP in the HCC remained unclear. In the present study, we revealed that OIT3 might function as a tumor suppressor gene that inhibits the proliferation, migration, and invasion abilities of HCC cell lines *in vitro* and *in vivo*. Furthermore, OIT3 promotes the ferroptosis of HCC cells by regulating the arachidonic acid metabolism and ROS accumulation, which is consistent with the previous observation suggesting that OIT3 is involved in lipid metabolism in the liver, including the triacylglycerol (TG) and very-low-density lipoproteins (VLDLs) (27). Yet, our results also showed that OIT3 mainly regulates the metabolism of arachidonic acid (AA), a classical type of PUFAs, thus adding a novel insight into the complicated lipid metabolism regulation mechanism of OIT3. Moreover, although the aberrant expression of OIT3 in HCC has been previously reported, the present study is the first to report its effect on ferroptosis induction. However, our findings are not consistent with data suggesting that LZP did not affect the cell growth in SMMC-7721 and L02 (24). This may be because SMMC-7721 was not considered an HCC cell line for the cross-contamination with other cells according to the HEPATOLOGY author guidelines, and the L02 was not an HCC cell line.

Previous studies reported that AA was a kind of n-6 long-chain PUFAs, anchoring in the phosphatidylethanolamine. As AA is the foremost target of LOXs, AA acts as an essential reaction substrate for ferroptosis (27, 34, 40, 41). AA can result in lipid peroxidation when residing in the high ROS tumor environment, which decreases the glutathione (GSH) level, acting as the “deadly sign” of ferroptosis (42). AA also composes a highly organized oxygenation center that might undergo severe peroxidation with the iron-dependent mechanism and formation of ROS, thus navigating cells to ferroptosis in gastric cancer cells (43). The auto-oxidation of AA could even contribute to lipid peroxidation in the acetaminophen-induced ferroptosis process in liver tissue (44). Accordingly, inhibiting the synthesis of AA could have a protective effect on ferroptosis (45). AA has been identified as the direct precursor of various types of bioactive mediators, including leukotrienes (LTs), such as LTB4, LTC4, and so on. The ferroptosis augmented by AA could be facilitated to reverse the drug resistance of cancer cells (42). It is noteworthy that the enzymatic oxidation of PUFAs could be selectively and specifically mediated by lipoxygenases (LOXs), including ALOX15, which contains iron in the catalytic region to stimulate the deoxygenation of PUFAs (46). ALOX15 can induce enzymatic production of a5-HOO-AA-PE (arachidonic acid-phosphatidylethanolamine), then mediate the oxidative cleavage of these initial HOO derivatives to proximate electrophiles, which in turn interact with the proteins, creating the plasma membranes’ pores (47, 48). ALOX15 has an important role in ferroptosis (49), as it promotes the generation of hydroperoxides in cellular membranes to induce ferroptosis in HT1080 cells (50). The oxidation of AA could be catalyzed by ALOX15 to produce 13-hydroperoxyoctadecadienoic acid and 15-hydroperoxyeicosatetraenoic acid, both of which were recognized as the intermediates for the formation of 4-HNE, a product of ferroptosis (51).

ALOX15 can metabolize AA in order to boost the generation of ROS and trigger ferroptosis (49). In this study, we found that upregulation of OIT3 increases the level of ALOX5 and promotes the arachidonic acid metabolism pathway, which was evidenced by the increased level of AA, TB4, LTC4, and LTD4, as well as LTE4 and is consistent with previous reports. Our results suggested that OIT3 functions as a tumor suppressor gene by mediating ferroptosis in HCC cells, thus providing a novel mechanism for the OIT3’s regulation in cancer progression. Nonetheless, the interaction between OIT3 and ALOX15 should be further investigated.

Moreover, besides the ALOX family, including ALOX15, AA could be metabolized by the CYP450 family (52). The cytochrome P450 was identified as pivotal for ferroptosis execution (53). CYP4F3, an enzyme of the CYP50 superfamily, generated ROS when metabolizing their substrates (54). The high level of intracellular ROS was identified as a key factor triggering ferroptosis. The association between cytochrome P450 activity and ROS production has been reported in various situations (55, 56). NADPH-cytochrome P450 reductase (POR) and NADH-cytochrome b5 reductase (CYB5R1) transfer electrons from NAD(P)H to oxygen, thus generating hydrogen peroxide, followed by reacting with iron to generate reactive hydroxyl radicals for PUFAs’ peroxidation and ferroptosis (57). Under the transcriptional modulation of the aryl hydrocarbon receptor (AhR), certain CYP450 superfamily members can be upregulated to increase the ROS level, and promote lipid peroxidation, thus finally inducing ferroptosis (58). CYP450 metabolizes the AA to produce 20−hydroxyeicosatetraenoic acid and increase NADPH oxidase activity, resulting in ROS elevation (58). Consistent with previous studies, our data revealed that OIT3 caused the increased expression of CYP4F3, the enhancement of AA’s metabolism, as well as striking accumulation of ROS in the HCC cells, following the interaction between the excessive ROS and AA to boost ferroptosis. Our findings provide further support for the premise that CYP4F3 epitomized a promotional effect of ferroptosis; however, the exact regulation mode of CYP4F3 on the AA metabolism is still not fully understood. Besides, future studies should further investigate how OIT3 upregulates the expression of CYP4F3.

## Conclusions

We found that OIT3 was downregulated in HCC tissues, and its expression level was significantly associated with clinical outcomes, suggesting it is a potential novel biomarker for the diagnosis and prognosis of HCC patients. Furthermore, OIT3 suppressed cell growth, migration, and invasion abilities in HCC cells *in vitro* and *in vivo*. This is the first study that demonstrated how OIT3 could upregulate the expression level of ALOX15 and CYP4F3 and then mediate the ROS accumulation and AA metabolism pathway activation, the key factors in triggering ferroptosis. To sum up, our data elucidated the regulation function of OIT3 in HCC and the corresponding mechanism. Given the pro-ferroptosis effect of OIT3 in HCC, targeting OIT3 could have therapeutic potential in clinical application.

## Data availability statement

The original contributions presented in the study are included in the article/**Supplementary Material**. Further inquiries can be directed to the corresponding authors.

## Ethics statement

The animal study was reviewed and approved by Peking University Third Hospital.

## Author contributions

JW: conceptualization, data curation, formal analysis, funding acquisition, investigation, writing-original draft, and writing-review and editing; AA: conceptualization, writing-review and editing, and funding acquisition; XY: conceptualization and writing-review and editing; YL, XZ, and GZ: formal analysis; SN and SH: funding acquisition; JL: conceptualization, writing-review and editing, and funding acquisition. All authors contributed to the article and approved the submitted version.

## Funding

This work was Funded by China Postdoctoral Science Foundation to JW (2020M683115), the National Natural Science Foundation of China (No. 81971719 to JL, 81903037 to SH, 81902909 to AA and 82102818 to JW), the Guangdong Medical Research Foundation (No. A2022121 to SN), the Major Science and Technology Program of Guangdong Province (No. 2020B0101130016 to JL) and the Guangzhou Key research and development Projects (No. 202103000021 to JL).

## Acknowledgments

We thank professor AA from Peking University Third Hospital for providing HepG2 and Huh7 cell lines.

## Conflict of interest

The authors declare that the research was conducted in the absence of any commercial or financial relationships that could be construed as a potential conflict of interest.

## Publisher’s note

All claims expressed in this article are solely those of the authors and do not necessarily represent those of their affiliated organizations, or those of the publisher, the editors and the reviewers. Any product that may be evaluated in this article, or claim that may be made by its manufacturer, is not guaranteed or endorsed by the publisher.

## Abbreviations

Hepatocellular carcinoma, HCC; Polyunsaturated fatty acids, PUFAs; Reactive oxygen species, ROS; Arachidonic acid, AA; Arachidonate-15-Lipoxygenase, ALOX15; Leukotriene B4, LTB4; OIT3-overexpressed lentivirus vector, OIT3-OE.
