# Corrigendum: OIT3 serves as a novel biomarker of hepatocellular carcinoma by mediating ferroptosis *via* regulating the arachidonic acid metabolism

**DOI:** 10.3389/fonc.2022.1038279

**Published:** 2022-10-26

**Authors:** Jie Wen, Abudureyimujiang Aili, Yao Xue Yan, YuLin Lai, Shaoqing Niu, Shasha He, Xiaokai Zhang, Guixiong Zhang, Jiaping Li

**Affiliations:** ^1^ Department of Interventional Oncology, The First Affiliated Hospital of Sun Yat-sen University, Guangzhou, China and Department of Medical Oncology and Radiation Sickness, Peking University Third Hospital, Beijing, China; ^2^ Department of Medical Oncology and Radiation Sickness, Peking University Third Hospital, Beijing, China; ^3^ Department of Dermatology, Peking University People’s Hospital, Beijing, China; ^4^ Deparment of Radiotherapy, The First Affiliated Hospital of Sun Yat-sen University, Guangzhou, China; ^5^ Department of Interventional Oncology, The First Affiliated Hospital of Sun Yat-sen University, Guangzhou, China

**Keywords:** HCC, OIT3, ferroptosis, arachidonic acid, biomarker

In the published article, there was an error in the author list, and authors Abudureyimujinag Aili, Yao Xue Yan were erroneously excluded from co-first authorship. Jie Wen, Abudureyimujiang Aili, Yao Xue Yan should be co-first authors. The corrected author list appears below.

Jie Wen^1*†^, Abudureyimujiang Aili^2†^, Yao Xue Yan^3†^, YuLin Lai^4^, Shaoqing Niu^4^, Shasha He^4^, Xiaokai Zhang^5^, Guixiong Zhang^5^ and Jiaping Li^5*^

^†^These authors have contributed equally to this work and share first authorship.

In the published article, there was an error in the Funding statement. There was a grammatical error and two funders were missed. The correct Funding statement appears below.

This work was Funded by China Postdoctoral Science Foundation to Jie Wen (2020M683115), the National Natural Science Foundation of China (No. 81971719 to Jiaping Li, 81903037 to Shasha He, 81902909 to Abudureyimujiang Aili and 82102818 to Wenjie), the Guangdong Medical Research Foundation (No. A2022121 to Shaoqing Niu), the Major Science and Technology Program of Guangdong Province (No. 2020B0101130016 to Jiaping Li) and the Guangzhou Key research and development Projects (No. 202103000021 to Jiaping Li).

In the published article, there was an error in the description of Malondialdehyde (MDA) measurement. A correction has been made to **Methods,** *Malondialdehyde (MDA) measurement*, line 239-245 in Paragraph 4 of Page 6. This sentence previously stated:

“The calculation method was as follows:

Δod was as follows calculation m

Δod was as follows calculation m

Δod waA600 (sample)-A600 (blank)

MDA (nmol/mgprot) =5 × 12.9ot) =55) =5t) =5A600 (blankSpr (sample protein concentration, 232 mg/mL).”

The corrected sentence appears below:

“ΔA450=A450 (sample)-A450 (blank)

ΔA532=A532 (sample)-A532 (blank)

ΔA600=A600 (sample)-A600 (blank)

MDA (nmol/mgprot) =5×(12.9×(ΔA532-ΔA600)-2.58×ΔA450)÷Spr (sample protein concentration, mg/mL).”

The authors apologize for these errors and state that they do not change the scientific conclusions of the article in any way. The original article has been updated.

## Publisher’s note

All claims expressed in this article are solely those of the authors and do not necessarily represent those of their affiliated organizations, or those of the publisher, the editors and the reviewers. Any product that may be evaluated in this article, or claim that may be made by its manufacturer, is not guaranteed or endorsed by the publisher.

