# Supplementary material for: OIT3 serves as a novel biomarker of hepatocellular carcinoma by mediating ferroptosis via regulating the arachidonic acid metabolism
Source: Front Oncol. 2022 Sep 5;12:977348. doi: 10.3389/fonc.2022.977348 (PMC9483180; doi:10.3389/fonc.2022.977348)
Supplement: Supplementary file 1 [file DataSheet_1.docx]

Supplementary Material

# Supplementary Figures and Tables

## Supplementary Tables

**Table S1.** The detailed information of antibodies used in the research

| Antibody NAME | Brand | Application and Condition | Cat number | Clone |
| --- | --- | --- | --- | --- |
| OIT3 | Abbexa company | WB (1:1000)  IHC (1:200) | abx103361 |  |
| GPX4 | Cell Signaling Technology company | WB (1:1000) | #52455 |  |
| GPX4 | Proteintech company | IHC (1:800) | 67763-1-Ig | 3F5G5 |
| GAPDH | Proteintech company | WB (1:10000) | 60004-1-Ig | 1E6D9 |
| CYP4F3 | Bioss company | WB (1:1000)  IHC (1:100) | bs-14160R |  |
| ALOX15 | Bioss company | WB (1:1000)  IHC (1:500) | bs-34007R |  |

**Table S2.** The top 20 significantly changing genes in five GSE databases

| GSEs  Genes | GSE45050 | GSE121248 | GSE45267-elder patients | GSE45267-young patients | GSE33006 |
| --- | --- | --- | --- | --- | --- |
| The top  20 significantly changing genes | CLEC1B | CXCL14 | CLEC1B | LINC01093 | MAGEA6 |
|  | IDO2 | IGFALS | CLEC4M | CNDP1 | MAGEA2B///MAGEA2 |
|  | GLS2 | ANGPTL6 | CLEC4G | NAT2 | LIN28B |
|  | BMP10 | VIPR1 | FCN2 | CYP2B6 | CLEC4M |
|  | CLEC4M | HHIP | HHIP | NEK2 | LOC101928916///NNMT |
|  | CLEC4G | CLEC1B | OIT3 | TTC36 | INS-IGF2///IGF2 |
|  | STAB2 | CDHR2 | FCN3 | LINC00844 | PCDH9 |
|  | NTF3 | CLEC4M | STAB2 | FLVCR1 | SDS |
|  | NRG1 | KCNN2 | CRHBP | TTK | LCAT |
|  | OIT3 | ECM1 | RSPO3 | LPA | P3H2 |
|  | SLCO4C1 | OIT3 | ECM1 | FCN2 | MME |
|  | ECM1 | FCN2 | CXCL14 | ASPM | C9 |
|  | BMPER | CCBE1 | PRC1 | STEAP4 | CES4A |
|  | CNTN3 | ADAMTS13 | ECT2 | GMNN | OIT3 |
|  | UROC1 | PLVAP | RACGAP1 | LINC01554 | CRHBP |
|  | HHIP | CLEC4G | PLAC8 | GLYAT | DCN |
|  | CXCL14 | ZGPAT | MARCO | CLEC1B | CFHR3 |
|  | CDH19 | ANKRD55 | CFP | OIT3 | COX7B2 |
|  | NRG1 | STAB2 | CCNB1 | FANCI | LOC105372969 |
|  | ABCA10 | CYP26A1 | PAMR1 | CLEC4G | MAGEA12 |

**Table S3.** Clinical characteristics of patients whose *OIT3* expression was evaluated in cancer tissues and adjacent tissues

| Clinicopathological variable | Value |
| --- | --- |
| Median age, years (range) | 52 (31-78) |
| Sex |  |
| Male | 80 (89.9%) |
| Female | 9 (10.1%) |
| T stage |  |
| T1 | 58 (65.2%) |
| T2 | 28 (31.4%) |
| T3 | 3 (3.4%) |
| AJCC stage |  |
| AJCC 1 | 58 (65.2%) |
| AJCC 2 | 29 (32.6%) |
| AJCC 3 | 2 (2.2%) |
| Recurrence |  |
| Negative | 41 (46.1%) |
| Positive | 48 (53.9%) |
| HBS infection |  |
| HBS(+) | 70 (78.7%) |
| HBS(-) | 19 (21.3%) |
| Total bilirubin (TB) |  |
| TB≤21 | 75 (84.3%) |
| TB＞21 | 14 (15.7%) |
| Alanine Aminotransferase (ALT, U/L) |  |
| ALT≤40 | 52 (58.4%) |
| ALT＞40 | 37 (41.6%) |
| Alpha-fetoprotein(AFP,μg/L) |  |
| AFP≤25 | 38 (42.7%) |
| AFP＞25 | 51 (57.3%) |
| Tumor size |  |
| ≤5cm | 62 (69.7%) |
| ＞5cm | 27 (30.3%) |
| OIT3 expression |  |
| OIT3 Low | 31 (34.8%) |
| OIT3 High | 58 (65.2%) |

**Table S4.** The average optical density (AOD) of the HCC tissues and corresponding para-cancerous tissue in microarray.


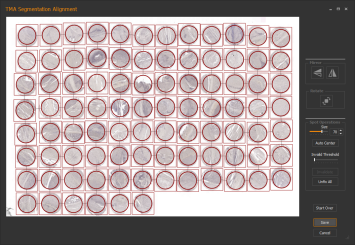


| Name | AOD  (HCC tissue) | AOD  (para-cancerous tissue) | Name | AOD  (HCC tissue) | AOD  (para-cancerous tissue) | Name | AOD  (HCC tissue) | AOD  (para-cancerous tissue) |
| --- | --- | --- | --- | --- | --- | --- | --- | --- |
| A1 | 0.076771 | 0.098000 | C9 | 0.079895 | 0.109100 | F5 | 0.081889 | 0.086700 |
| A2 | 0.073991 | 0.108100 | C10 | 0.085772 | 0.087800 | F6 | 0.08299 | 0.097900 |
| A3 | 0.066586 | 0.108200 | C11 | 0.077102 | 0.109700 | F7 | 0.084239 | 0.098000 |
| A4 | 0.067597 | 0.108300 | C12 | 0.075424 | 0.097200 | F8 | 0.07807 | 0.087400 |
| A5 | 0.074684 | 0.082000 | D1 | 0.086045 | 0.109780 | F9 | 0.086783 | 0.099200 |
| A6 | 0.082292 | 0.087000 | D2 | 0.079504 | 0.109720 | F10 | 0.088657 | 0.098000 |
| A7 | 0.086883 | 0.109300 | D3 | 0.081497 | 0.109710 | F11 | 0.079481 | 0.108100 |
| A8 | 0.073999 | 0.086900 | D4 | 0.083348 | 0.088000 | F12 | 0.083191 | 0.108200 |
| A9 | 0.074128 | 0.089000 | D5 | 0.073872 | 0.108710 | G1 | 0.073359 | 0.108300 |
| A10 | 0.067832 | 0.088000 | D6 | 0.081015 | 0.097200 | G2 | 0.072282 | 0.082000 |
| A11 | 0.083736 | 0.087000 | D7 | 0.085754 | 0.108770 | G3 | 0.079482 | 0.087000 |
| A12 | 0.082614 | 0.088000 | D8 | 0.07734 | 0.086300 | G4 | 0.071606 | 0.109300 |
| B1 | 0.075389 | 0.108650 | D9 | 0.089458 | 0.086900 | G5 | 0.073219 | 0.086900 |
| B2 | 0.077125 | 0.118500 | D10 | 0.075395 | 0.108680 | G6 | 0.095865 | 0.089000 |
| B3 | 0.079758 | 0.087500 | D11 | 0.093129 | 0.087200 | G7 | 0.076153 | 0.088000 |
| B4 | 0.085996 | 0.089000 | D12 | 0.067882 | 0.108740 | G8 | 0.080434 | 0.087000 |
| B5 | 0.077845 | 0.080000 | E1 | 0.083858 | 0.108910 | G9 | 0.077953 | 0.088000 |
| B6 | 0.078901 | 0.084000 | E2 | 0.076867 | 0.087700 | G10 | 0.07471 | 0.108650 |
| B7 | 0.077803 | 0.080000 | E3 | 0.082841 | 0.087200 | G11 | 0.075712 | 0.118500 |
| B8 | 0.085317 | 0.086000 | E4 | 0.076445 | 0.108750 | G12 | 0.068361 | 0.087500 |
| B9 | 0.077726 | 0.089000 | E5 | 0.081294 | 0.087100 | H1 | 0.074138 | 0.089000 |
| B10 | 0.09497 | 0.095000 | E6 | 0.103224 | 0.087600 | H2 | 0.07268 | 0.080000 |
| B11 | 0.085642 | 0.118800 | E7 | 0.078674 | 0.096800 | H3 | 0.079126 | 0.084000 |
| B12 | 0.084029 | 0.109400 | E8 | 0.081042 | 0.127900 | H4 | 0.080174 | 0.080000 |
| C1 | 0.076716 | 0.108800 | E9 | 0.080335 | 0.180000 | H5 | 0.085143 | 0.086000 |
| C2 | 0.076979 | 0.087200 | E10 | 0.085345 | 0.097200 | H6 | 0.087296 | 0.089000 |
| C3 | 0.082429 | 0.109680 | E11 | 0.081736 | 0.109790 |  |  |  |
| C4 | 0.070176 | 0.128900 | E12 | 0.084616 | 0.109850 |  |  |  |
| C5 | 0.079177 | 0.108690 | F1 | 0.082005 | 0.109710 |  |  |  |
| C6 | 0.08946 | 0.109790 | F2 | 0.074457 | 0.087600 |  |  |  |
| C7 | 0.078144 | 0.087200 | F3 | 0.088826 | 0.087500 |  |  |  |
| C8 | 0.085949 | 0.109780 | F4 | 0.084484 | 0.097100 |  |  |  |

**Table S5.** Associations between OIT3 expression and clinicopathological features

| Clinicopathological variable | OIT3 Low | OIT3 High | χ2 value | P-value |
| --- | --- | --- | --- | --- |
| Sex |  |  | 0.4076 | 0.523 |
| Male | 27 | 53 |  |  |
| Female | 4 | 5 |  |  |
| Age at diagnosis, years |  |  | 1.4529 | 0.228 |
| ＜60 | 21 | 46 |  |  |
| ≥60 | 10 | 12 |  |  |
| T stage |  |  | 7.7487 | **0.021** |
| T1 | 16 | 42（72.41%） |  |  |
| T2 | 12 | 16（57.14%） |  |  |
| T3 | 3 | 0（0.00%） |  |  |
| AJCC stage |  |  | 6.3598 | **0.042** |
| AJCC I | 16 | 42（72.41%） |  |  |
| AJCC II | 13 | 16（55.17%） |  |  |
| AJCC III | 2 | 0（0.00%） |  |  |
| Recurrence status |  |  | 5.5561 | **0.018** |
| Free | 9 | 32（78.05%） |  |  |
| Positive | 22 | 26（54.17%） |  |  |
| HBS infection |  |  | 0.1126 | 0.737 |
| HBS(+) | 25 | 45 |  |  |
| HBS(-) | 6 | 13 |  |  |
| Total bilirubin (TB) |  |  | 2.4249 | 0.119 |
| TB≤21 | 21 | 51 |  |  |
| TB＞21 | 8 | 6 |  |  |
| Alanine Aminotransferase (ALT, U/L) |  |  | 0.0026 | 0.956 |
| ALT≤40 | 18 | 34 |  |  |
| ALT＞40 | 13 | 24 |  |  |
| Alpha-fetoprotein(AFP,μg/L) |  |  | 1.0115 | 0.315 |
| AFP≤25 | 11 | 27 |  |  |
| AFP＞25 | 20 | 31 |  |  |
| Tumor size |  |  | 1.5779 | 0.209 |
| ≤5cm | 19 | 43 |  |  |
| ＞5cm | 12 | 15 |  |  |

**Table S6.** Results of univariate analysis of potential patient characteristics influencing OS and DFS

| Parameter | P-value (OS) | P-value (DFS) |
| --- | --- | --- |
| Sex | 0.137 | 0.211 |
| Age | 0.185 | 0.179 |
| T stage | **0.036** | **0.035** |
| AJCC stage | **0.046** | **0.039** |
| Recurrence status | **0.000** | **0.000** |
| HBS infection | 0.900 | 0.567 |
| TB | 0.396 | 0.491 |
| ALT | 0.257 | 0.344 |
| AFP | 0.219 | 0.266 |
| Tumor size | 0.148 | **0.040** |
| OIT3 expression | **0.005** | **0.001** |

Notes: T stage, AJCC stage, and Recurrence status were significantly related to the OS and DFS of HCC patients, while tumor size was only significantly related to the DFS. Abbreviations: OS, overall survival; DFS, disease-free survival; TB, total bilirubin; ALT, alanine aminotransferase; AFP, alpha-fetoprotein. 0.000 means P < 0.001.

**Table S7.** Results of multivariate analysis of potential patient characteristics influencing OS and DSS

| Parameter | P-value  (OS) | Hazard ratio  (OS) | 95 % CI  (OS) | P-value  (DFS) | Hazard ratio  (DFS) | 95 % CI  (DFS) |
| --- | --- | --- | --- | --- | --- | --- |
| T stage | 0.976 |  |  | 0.496 |  |  |
| AJCC stage | 0.911 |  |  | 0.582 |  |  |
| Recurrence status | **0.000** | 7.825 | (3.404,17.992) | **0.000** | 166.575 | (19.834,1399.009) |
| OIT3 expression | 0.137 |  |  | **0.033** | 0.493 | (0.257,0.946) |
| Tumor size |  |  |  | 0.825 |  |  |

Notes: Recurrence status was the independent prognostic factor of the OS and DFS of HCC patients, while *OIT3* expression was the independent prognostic factor of DFS of HCC patients. Using the recurrence-free as the reference, patients with recurrence had a poor clinical outcome (OS: HR=7.825, 95%CI=3.404-17.992; DFS: HR=166.575, 95%CI =19.834-1399.009). Compared to patients with low OIT3 expression, those with high OIT3 expression had a good clinical outcome (DFS: HR=0.493, 95%CI=0.257-0.946). Abbreviations: OS, overall survival; DFS, disease-free Survival. 0.000 means P < 0.001.

## Supplementary Figures


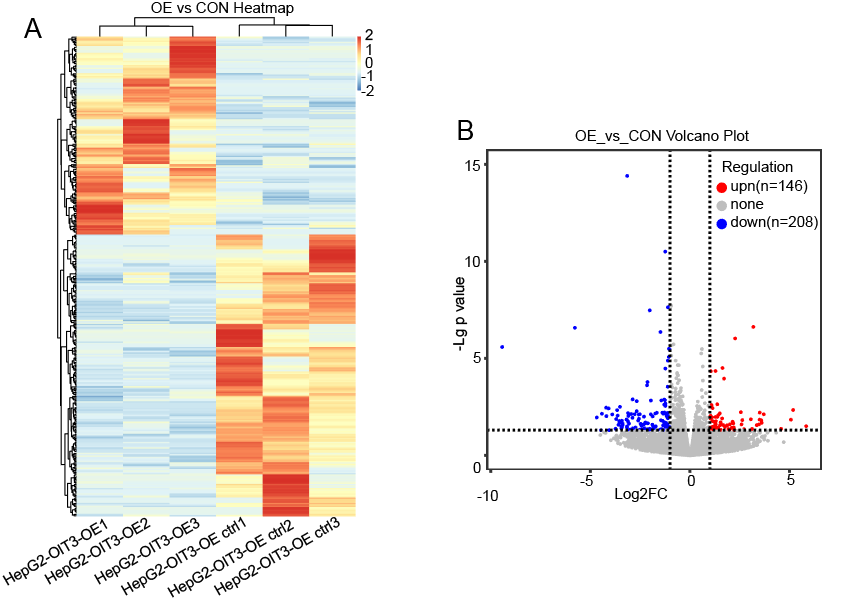


**Fig S1. The expression analysis for the RNA-sequence.** (A) The heat map showed the expression pattern of HepG2 cells with different OIT3 levels. (B) The volcano plots present the differently expressed genes of HepG2 cells with different OIT3 levels. As shown, OIT3 significantly upregulated 146 genes and downregulated 208 genes in HepG2 cells. Abbreviations: OIT3-OE or OE, OIT3-overexpressed lentivirus vector; OIT3-OE ctrl or ctrl, OIT3-overexpressed lentivirus vector control.


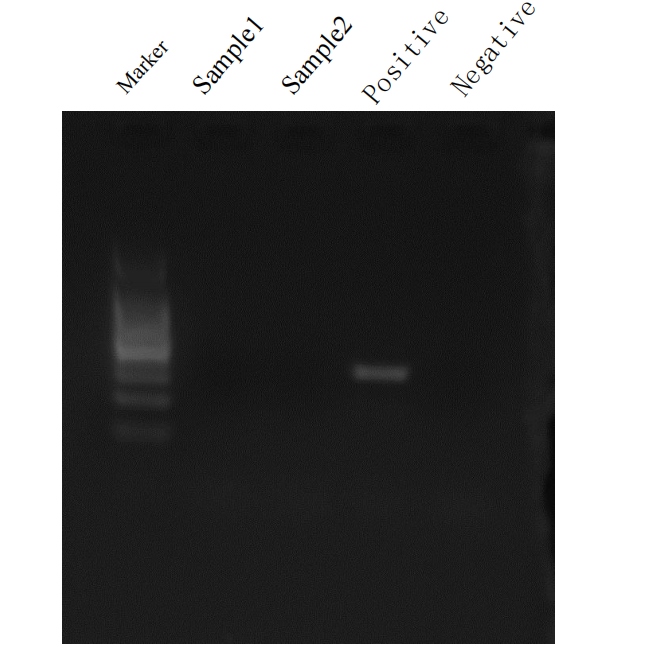


Sample 1：The cell culture supernatant of HepG2

Sample 2：The cell culture supernatant of Huh7

Positive：Positive control

Negative：Negative control

Marker(From bottom to top):100、200、300、400、500、600、700bp

**Fig S2.The mycoplasma test result of cell culture supernatant of HepG2 and Huh7.** 350bp bands can be seen in the positive control lane, and no corresponding bands can be found in sample 1-2 and negative control, so sample 1-2 is determined to be negative.


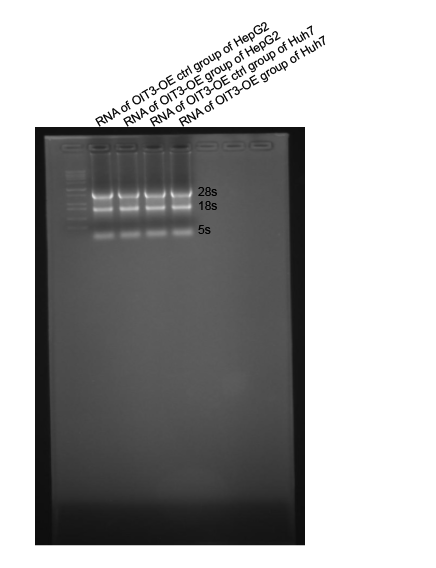


**Fig S3. The RNA QC of samples in different groups by integrity test using electrophoresis.**

**
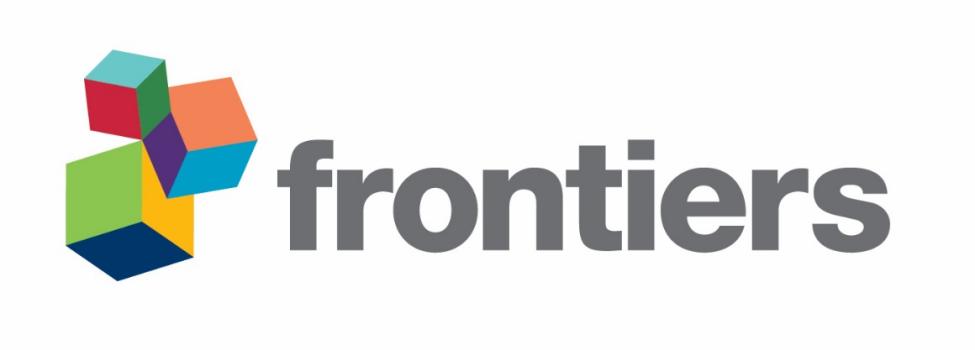
**
